# Vitamin D Deficiency among Adults with History of Pulmonary Tuberculosis in Korea Based on a Nationwide Survey

**DOI:** 10.3390/ijerph14040399

**Published:** 2017-04-10

**Authors:** Mi Hyun Joo, Mi Ah Han, Sun Mi Park, Hwan Ho Shin

**Affiliations:** 1 Department of Preventive Medicine, College of Medicine, Chosun University, Gwangju 61452, Korea; jsm0417@hanmail.net (M.H.J.); holasm@hanmail.net (S.M.P.); ggamjang3@naver.com (H.H.S.); 2 Department of Health Science, Graduate School, Chosun University, Gwangju 61452, Korea

**Keywords:** nutrition surveys, tuberculosis, vitamin D, vitamin D deficiency

## Abstract

We investigated the prevalence of vitamin D deficiency among individuals who have a history of tuberculosis (TB) diagnosis in Korea. Using the 5th Korean National Health and Nutrition Examination Survey, we selected 805 individuals with a history of TB diagnosis and 16,049 controls without a history of TB. Vitamin D deficiency was defined as a 25(OH)D level less than 20 ng/mL. Vitamin D deficiency was revealed in 71.7% of the individuals with a history of TB diagnosis and in 72.1% of the controls. Vitamin D deficiency was more likely in women than in men, in people who engaged in other jobs or were unemployed than in people who engaged in skilled agricultural, forestry, and fishery jobs, and in people who walked 3–5 days per week than in people who walked 6–7 days per week. Vitamin D deficiency was highly prevalent in the TB group. Regular examination and strategies to increase vitamin D levels in individuals with a history of TB are needed, as vitamin D is associated with TB conditions and bone disease.

## 1. Introduction

Tuberculosis (TB) is an infectious disease caused by *Mycobacterium tuberculosis* and is a serious problem around the world. In 2014, the total number of TB cases in the world stood at 9.6 million, and the number of TB-related deaths was 1.5 million [1]. According to the World Health Organization’s 2015 Global Tuberculosis Report, the Korean incidence of TB in 2014 was 86/100,000 individuals, and that incidence of TB takes the top spot among the member nations of the Organization for Economic Cooperation and Development. TB remains the highest public health priority in Korea [2,3].

Vitamin D is an important hormone for skeletal growth and maintenance. It maintains mineral homeostasis and plays an important role in various tissues [4]. Vitamin D is produced naturally from the skin if the skin is exposed to the sun. Vitamin D produced from the skin is prone to deficiency nowadays due to modern lifestyles. Vitamin D deficiency has been reported to be associated with cancer [5] as well as chronic diseases like hypertension [6], autoimmune disease [7], and diabetes [8]. Hence, there has recently been a growing interest in vitamin D around the globe. Few reports have examined the prevalence of vitamin D deficiency among TB patients. A case-control study of 105 TB patients and 255 controls reported that vitamin D deficiency was found in 57% of TB patients and 33% of controls [9]. In a different case-control study in Pakistan, vitamin D deficiency was common among TB patients (118/260, 45%) and was independently associated with susceptibility to TB (adjusted odds ratio (aOR) =1.87, 95% confidence interval (CI) = 1.15–3.04) [10]. In another cross-sectional study in northern India, patients with pulmonary TB had significantly lower mean vitamin D levels (11.2 ± 6.5 ng/mL) and had the highest prevalence of vitamin D deficiency (92%) compared with other groups [11].

The prevalence of vitamin D deficiency has not been adequately determined among Korean subjects with a history of TB. Therefore, we investigated the prevalence of vitamin D deficiency among individuals who have a history of TB diagnosis in Korea. We also investigated the difference between subjects with TB history and controls and identified the factors associated with vitamin D deficiency among TB patients.

## 2. Materials and Methods

### 2.1. Data Source

This study was based on data acquired in the 5th (2010–2012) Korean National Health and Nutrition Examination Survey (KNHANES). The KNHANES is a nationwide survey that investigates the health status, health behavior, and nutritive conditions of the Korean people. KNHANES consisted of three components: health interview, health examination and nutrition survey. A stratified, multistage probability sampling design was used to interview household units. The KNHANES response rates were 81.9% (8958 of 10,938) in 2010, 80.4% (8518 of 10,589) in 2011, and 80.0% (8058 of 10,069) in 2012. Among a total of 25,534 participants, 24,173 subjects participated in health interview and health examination. Among them, participants ≤19 years of age (n = 5602), participants with missing data of TB status or serum 25(OH)D level (n = 1474) and covariates (n = 243) were excluded. Finally, a total of 16,854 adults with recorded serum 25(OH)D levels were included in the study. All data were approved by the Institutional Review Board of the Korea Centers for Disease Control (2010-02CON-21-C, 2011-02CON-06-C and 2012-01EXP-01-2C). Informed consent was received from each participant before the survey [12].

### 2.2. History of Tuberculosis Diagnosis

Participants with a history of TB diagnosis were defined as those who answered “yes” to the question, “Have you ever heard from a doctor that you had TB?” Those who had a history of TB diagnosis were asked about their age at diagnosis. The time since diagnosis was calculated from the difference between the age at diagnosis and the age at survey participation. Controls were defined as subjects who reported no history of TB diagnosis.

### 2.3. Vitamin D Deficiency

Blood samples were separated and refrigerated. 25(OH)D levels were measured by radioimmunoassay methods using a 1470 WIZARD gamma-Counter (PerkinElmer, Turku, Finland). Vitamin D deficiency was defined as a 25(OH)D level <20 ng/mL as per the guideline proposed by the Institute of Medicine.

### 2.4. Covariates

Several variables such as sex, age (19–44, 45–64, ≥65 years), education (≤high school, ≥college), monthly income (low, middle, high), occupation type (skilled agricultural, forestry, fishery workers; others; unemployed (e.g., housewife, student)), smoking status (current smoker, ex-smoker, non-smoker), drinking status (current drinker, ex-drinker, non-drinker), subjective health status (good, fair, bad), body mass index (underweight, normal, overweight), walking in a week (0–2, 3–5, 6–7 days), and the use of dietary supplements (yes, no) were used.

### 2.5. Statistical Analysis

Statistical analysis was conducted using SAS Version 9.3 (SAS Institute INC, Cary, NC, USA). All analyses were conducted using survey weighting to account for the multistage probability sampling design. The general characteristics of the subjects by history of TB diagnosis were shown as the number and percentage and compared using chi-square tests. The prevalence of vitamin D deficiency by the subjects’ characteristics in the TB and control groups was tested with chi-square tests. Multiple logistic regression analyses were used to identify factors associated with vitamin D deficiency in the subjects with a history of TB diagnosis and in the non-TB controls. Numbers were expressed as crude numbers, and all other values were expressed as weighted values.

## 3. Results

### 3.1. General Characteristics of Subjects by History of TB Diagnosis

The general characteristics of the TB group and the controls were substantially different. The proportions of participants ≥65 years of age in the TB and control groups were 20.9% and 12.6%, respectively (*p* < 0.001). The most common age at diagnosis among those with a history of TB diagnosis was 19–44 years (55.8%), and the time since diagnosis was 20–39 years for 46.1% of the TB group (Table 1).

### 3.2. Prevalence of Vitamin D Deficiency in the TB and Control Groups

The mean vitamin D levels were 17.5 ng/mL, 17.6 ng/mL, and 17.5 ng/mL in the total sample, the TB group, and the controls, respectively. The rate of vitamin D deficiency (<20 ng/mL) was 72.0% among all the subjects. Vitamin D deficiency was found in 71.7% of the TB group and in 72.1% of the controls. Multiple logistic regression analysis showed that the prevalence of vitamin D deficiency was similar between the TB group and the controls after controlling for several covariates (aOR = 0.97, 95% CI = 0.79–1.18).

### 3.3. Factors Associated with Vitamin D deficiency in the TB and Control Groups

The prevalence of vitamin D deficiency was significantly higher among women (80.4%), younger subjects (79.2%), subjects whose type of job was “other” (75.8%), and non-smokers (77.0%). These figures were similar to those identified in the non-TB controls (Table 2).

Multiple logistic regression analysis showed that women had a significantly higher aOR for vitamin D deficiency within the TB group (aOR = 2.37, 95% CI = 1.04–5.40). TB subjects whose type of job was ”other” (aOR = 3.40, 95% CI = 1.51–7.66), were unemployed (aOR = 2.89, 95% CI = 1.34–6.23), and who walked 3–5 days per week (aOR = 1.80, 95% CI = 1.05–3.10) were more likely to have vitamin D deficiency. The use of dietary supplements and the time since TB diagnosis were not associated with vitamin D deficiency (Table 3).

Among the non-TB controls, sex, age, education, occupation, smoking status, drinking status, walking in a week, and the use of dietary supplements were associated with vitamin D deficiency (Table 3).

## 4. Discussion

A meta-analysis of in vivo studies showed that low serum vitamin D levels were associated with a higher risk of active TB [13]. Decreased vitamin D levels in patients with latent TB were associated with a 5-fold increased risk of progression to TB [14,15]. We investigated the prevalence of vitamin D deficiency and examined the factors associated with vitamin D deficiency among individuals with TB in Korea. Approximately 71.7% and 72.1% of the TB group and the controls, respectively, exhibited vitamin D deficiency. The prevalence and associated factors were also compared in subjects without a history of TB. Sex, occupation, and number of days of walking per week were associated with vitamin D deficiency in the TB group in multiple analyses.

Vitamin D deficiency can be associated with TB due to its essential functions in the immune system. Vitamin D can influence on TB through the regulation of cathelicidin which has a direct antibacterial effect on TB [16] and vitamin D receptor gene polymorphisms was associated with susceptibility to TB [17]. Vitamin D modulates immune functions mediated by monocytes, macrophages, dendritic cells, T cells, B cells [18].

In our study, the prevalence of vitamin D deficiency was high in both the TB group and the controls (71.7% and 72.1%, respectively). A previous case-control study of 362 TB patients and 494 controls revealed that vitamin D deficiency was more common in TB patients (46.0% vs. 39%) [19]. The high prevalence of vitamin D deficiency that we found in both groups suggests that vitamin D deficiency is not a problem limited to individuals with specific conditions such as TB but is rather a general public health burden in Korea. Recent reviews emphasizing the high prevalence of vitamin D deficiency is a global problem [20,21]. The prevalence of vitamin D deficiency (<20 ng/mL) was estimated to be 36% in USA, 61% in Canada, 57%–64% in Europe and 78%–98% in Asia [20]. Vitamin D deficiency is prevalent in Asia including Korea. Latitude of the countries, attitudes and behavior toward sunlight exposure were the major determinants of vitamin D status [22].

A high prevalence of vitamin D deficiency in women than in men was revealed (80.4% vs. 65.6%) in the TB group. Vitamin D deficiency was most prevalent in subjects 19–44 years of age (79.2%) and was least prevalent in subjects over 65 years of age (57.6%). Vitamin D deficiency is highly prevalent and greatly contributes to women’s health [23] and young and middle-aged adults have high risks for vitamin D deficiency worldwide because of lifestyle changes such as decreased outdoor activity and the avoidance of sun exposure [22,24]. Generally, men seem to be more affected than women by TB [25]. But according to age group there was a higher risk for younger women than men [26,27]. Our high prevalence of vitamin D deficiency in women and younger subjects who were high risk groups for TB suggested that management of vitamin D status and further studies on the association between TB and vitamin D are needed in these group.

In terms of occupation, unemployed subjects had relatively high rates of vitamin D deficiency in TB group. In our study, the unemployment rate in TB groups was higher than controls (41.0% vs. 34.3%). Unemployment among TB patients was associated with poor course of the disease and was a serious problem [28,29]. Strategies and efforts are needed to improve the occupational status among TB subjects. Due to the nature of KNHANES, we did not directly assess sun exposure or outdoor activities. We considered skilled agricultural, forestry, and fishery work as outdoor activities and other jobs or unemployment as indoor activities. Outdoor activities were associated with lower risk of vitamin D deficiency [30,31].

The use of dietary supplements was associated with a lower risk of vitamin D deficiency among the controls but not among the subjects with a history of TB diagnosis. Vitamin D supplementation is usually prescribed for osteoporosis prevention, yet guidelines for its use to prevent TB are deficient, despite the rarity of adverse effects [32]. Experts recommend the maintenance of an appropriate vitamin D concentration (30–44 ng/mL) to prevent vitamin D-related illness [33,34] and suggest taking vitamin D 800–1000 International Unit/day [35]. In a recent review, several studies reported that vitamin D supplementation has benefited TB patients by several means including the shortening of the sputum conversion period and improved inhibitory effects on monocyte counts and inflammatory cytokines. However, several other studies reported no significant associations. It is possible that the association could be influenced by the vitamin D dose and seasonality [16]. Unfortunately, the KNHANES did not record the type and amount of supplements. Further studies with sufficient vitamin D supplementation are needed to define the role of vitamin D in TB.

Our study has some limitations. First, we used a cross-sectional design, so we could not identify a causal relationship between vitamin D deficiency and TB. Second, the KNHANES did not record the current status of TB and associated clinical data, and it excluded institutionalized individuals and TB patients who were admitted to the hospital or were convalescing. Therefore, the TB group in our study might not be representative of all individuals who have a history of TB diagnosis in Korea. Also, KNHANES could not distinguish between latent or active TB. The history of TB was measured by self-reported method, and positive TB history could be interpreted as having active or latent TB. Clinical significance and its health burden are substantially different according to TB status (active or latent). Previous studies reported that vitamin D associated with both active or latent TB infection and vitamin D deficiency was associated with the risk of active TB in subjects with latent TB infection [36,37]. On the other hand, vitamin D level was not different between latent TB and controls groups [38]. TB status (active or latent) should be considered in further study about the effect of vitamin D on TB. Third, the serum vitamin D level is influenced by seasonality, and we could not use the information about the survey time. The KNHANES data are collected throughout the year, however, which should solve the problem of seasonal variations [12]. Fourth, history of TB diagnosis was based on self-report and there was a risk of recall bias. Fifth, there were significant differences in general characteristics (such as gender, age group, occupation, etc.) of TB groups and controls. These differences can compromise the comparison between TB group and controls. Matched controls or subgroup analysis according to covariates might be needed to solve these differences.

Vitamin D deficiency was highly prevalent in the TB group, and the prevalence was similar to that among the non-TB controls in Korea. Regular examination and strategies to elevate vitamin D levels in individuals with a history of TB diagnosis are needed, because vitamin D is associated with TB conditions and bone disease.

## 5. Conclusions

Vitamin D deficiency was highly prevalent in the TB group, and the prevalence was similar to that among the non-TB controls in Korea. Regular examination and strategies to elevate vitamin D levels in individuals with a history of TB diagnosis are needed, because vitamin D is associated with TB conditions and bone disease.

## Figures and Tables

**Table 1:** General characteristics of subjects by history of TB diagnosis.

Characteristics	Self-Reported History of TB (n = 805)	No-Reported History of TB (n = 16,049)	*p*-Value
Sex			<0.0001
Male	442 (58.5)	6775 (49.5)	
Female	363 (41.5)	9274 (50.5)	
Age (years)			<0.0001
19–44	180 (30.5)	6592 (52.5)	
45–64	350 (48.6)	5941 (34.9)	
≥65	275 (20.9)	3516 (12.6)	
Education			0.375
≤High school	576 (69.3)	11,112 (67.3)	
≥College	229 (30.7)	4930 (32.7)	
Monthly income			0.913
Low	191 (27.4)	3819 (26.6)	
Middle	394 (49.6)	8025 (50.3)	
High	206 (23.0)	4050 (23.2)	
Occupation			0.006
Skilled agricultural, forestry, and fishery workers	61 (5.9)	1348 (6.7)	
Others	373 (53.0)	8301 (59.0)	
Unemployed	370 (41.0)	6355 (34.3)	
Smoking status			<0.0001
Current smoker	163 (27.2)	3337 (26.8)	
Ex-smoker	249 (29.5)	3174 (19.4)	
Non-smoker	393 (43.3)	9538 (53.8)	
Drinking status			0.124
Current drinker	136 (14.6)	2255 (12.0)	
Ex-drinker	565 (75.8)	11,638 (77.9)	
Non-drinker	104 (9.6)	2156 (10.0)	
Subjective health status			<0.0001
Good	223 (28.6)	5393 (34.9)	
Fair	380 (48.1)	7663 (48.4)	
Bad	202 (23.3)	2993 (16.7)	
Body mass index			<0.0001
Underweight	49 (7.2)	676 (4.5)	
Normal	585 (72.2)	10,103 (62.7)	
Overweight	170 (20.6)	5177 (32.8)	
Walking days per week			0.175
0–2	299 (37.5)	5500 (33.6)	
3–5	240 (29.3)	4932 (30.1)	
6–7	266 (33.3)	5614 (36.2))	
Dietary supplements			0.101
Yes	349 (46.5)	6564 (42.7)	
No	372 (53.5)	7685 (57.3)	
Age at diagnosis (years)			
19–29	327 (55.8)	-	
30–39	142 (18.4)	-	
≥40	197 (25.7)	-	
Time since diagnosis (years)			
<10	130 (19.9)	-	
10–19	133 (20.2)	-	
20–39	373 (46.1)	-	
≥40	169 (13.9)	-	

Data are expressed as crude numbers (weighted %).

**Table 2:** Prevalence of vitamin D deficiency by subject characteristics in the TB and control groups.

Characteristics	Self-Reported History of TB		No Reported History of TB	
	% with Vitamin D Deficiency	*p*-Value	% with Vitamin D Deficiency	*p*-Value
Total	71.7		72.1	
Sex		<0.001		<0.001
Male	65.6		66.2	
Female	80.4		77.8	
Age (years)		<0.001		<0.001
19–44	79.2		79.6	
45–64	73.2		65.4	
≥65	57.6		58.8	
Education		0.109		<0.001
≤High school	69.7		69.3	
≥College	76.3		77.8	
Monthly income		0.829		0.163
Low	71.3		70.9	
Middle	72.3		73.0	
High	69.1		71.2	
Occupation		<0.001		<0.001
Skilled agricultural, forestry, and fishery workers	37.2		41.1	
Others	75.8		73.7	
Unemployed	71.5		75.2	
Smoking status		0.016		<0.001
Current smoker	72.9		70.7	
Ex-smoker	63.0		63.3	
Non-smoker	77.0		75.9	
Drinking status		0.651		0.027
Current drinker	75.9		73.5	
Ex-drinker	71.1		72.3	
Non-drinker	70.8		68.5	
Subjective health status		0.356		0.429
Good	71.0		71.3	
Fair	74.3		72.6	
Bad	67.3		72.3	
Body mass index		0.575		<0.001
Underweight	72.9		79.9	
Normal	70.5		72.0	
Overweight	75.7		70.9	
Walking days per week		0.062		<0.001
0–2	73.8		72.3	
3–5	76.3		74.4	
6–7	65.4		69.9	
Dietary supplements		0.674		<0.001
Yes	69.7		68.6	
No	71.6		73.8	
Age at diagnosis (years)		0.183		
19–29	73.7			
30–39	66.8			
≥40	64.9			
Time since diagnosis (years)		0.289		
<10	78.5			
10–19	67.9			
20–39	71.8			
≥40	67.6			

Data are expressed as weighted %.

**Table 3:** Odds ratios (95% confidence interval) for vitamin D deficiency in the TB and control groups.

Characteristics	Self-Reported History of Tuberculosis	No-Reported History of Tuberculosis
Sex (Male)		
Female	2.37 (1.04–5.40)	1.93 (1.68–2.23)
Age (≥65)		
19–44	2.14 (0.84–5.47)	2.28 (1.93–2.71)
45–64	1.57 (0.88–2.77)	1.30 (1.13–1.50)
Education (≤High school)		
≥College	1.08 (0.60–1.97)	1.18 (1.04–1.34)
Monthly income (High)		
Low	0.95 (0.47–1.94)	0.94 (0.80–1.11)
Middle	1.11 (0.61–2.02)	1.07 (0.93–1.22)
Occupation/(Skilled agricultural, forestry, and fishery workers)		
Others	3.40 (1.51–7.66)	2.97 (2.35–3.75)
Unemployed	2.89 (1.34–6.23)	2.98 (2.36–3.77)
Smoking status (Current smoker)		
Ex-smoker	0.72 (0.36–1.46)	0.82 (0.70–0.96)
Non-smoker	0.64 (0.26–1.54)	0.94 (0.80–1.12)
Drinking status (Current drinker)		
Ex-drinker	0.94 (0.47–1.88)	0.85 (0.73–0.99)
Non-drinker	0.98 (0.41–2.32)	0.81 (0.65–0.99)
Body mass index (Normal)		
Underweight	1.10 (0.39–3.05)	1.05 (0.82–1.34)
Overweight	1.55 (0.84–2.89)	1.02 (0.91–1.14)
Walking days per week (6–7)		
0–2	1.38 (0.82–2.32)	1.12 (0.99–1.26)
3–5	1.80 (1.05–3.10)	1.19 (1.05–1.36)
Dietary supplements (Yes)		
No	1.47 (0.91–2.36)	1.49 (1.34–1.66)
Time since diagnosis (≥40 years)		
<10	1.11 (0.50–2.44)	
10–19	0.47 (0.21–1.07)	
20–39	0.64 (0.35–1.17)	
